# Making DEEP Sense of Lifestyle Risk and Resilience

**DOI:** 10.3389/fnagi.2019.00171

**Published:** 2019-07-17

**Authors:** Gerd Kempermann

**Affiliations:** ^1^German Center for Neurodegenerative Diseases (DZNE), Dresden, Germany; ^2^Center for Regenerative Therapies (CRTD) TU Dresden, Dresden, Germany

**Keywords:** prevention, Alzheimer’s disease, neurodegeneration, public health, personal medicine

## Abstract

To effectively promote life-long health and resilience against – for example – neurodegenerative diseases, evidence-based recommendations must acknowledge the complex multidimensionality not only of the diseases but also of personal lifestyle. In a straightforward descriptive and heuristic framework, more than 50 potential lifestyle factors cluster around diet (D), education (E), exercise (E), and purpose (P), unveiling their many relationships across domains and scales. The resulting systematics and its visualization might be a small but helpful step toward the development of more comprehensive, interdisciplinary models of lifestyle-dependent risk and resilience and a means to explain the opportunities and limitations of preventive measures to the public and other stakeholders. Most importantly, this perspective onto the subject implies that not all lifestyle factors are created equal but that there is a hierarchy of values and needs that influences the success of lifestyle-based interventions.

## Introduction

Lifestyle-based health interventions promise solutions to pressing health problems by providing broad access to prevention and healthy aging. Preventing dementia is a prime target of these ambitions. After hundreds of failed clinical trials for treatments of neurodegenerative disease, especially of Alzheimer’s disease (AD) (Cummings et al., 2014), the insight has grown that neither conventional pharmacological targets nor immune-based strategies alone will be sufficient to conquer the problem at large. Some trials have thus reformulated their goal from curing manifest pathology to secondary prevention (Livingston et al., 2017).

This step is wise, because despite the failing therapeutic trials, within age-cohorts the risk for AD has been decreasing over the past decades (Derby et al., 2017). This “success” cannot be attributed to specific therapeutic interventions. Given that genetic factors did not change during this period, the decrease indicates that, in sum, modifiable factors must have exerted a measurable positive impact. As Fries et al. (2011) already wrote in 2011: “*If we can accomplish morbidity compression without a strategy, as over the past thirty years, then we should be able to further improve if we have a plan.*” How might such plan be developed?

Given the complexity of the subject, one critical prerequisite is to first systematize the available knowledge. This is necessary, because “lifestyle factors” are a category without sharp boundaries and a common definition but with many stakeholder-specific connotations.

The proposed DEEP framework is a simple way to support, yet not replace, this process across domains, disciplines, and stakeholders. It consists of common language descriptors that in the absence of a unifying theory and a comprehensive model of lifestyle-dependent risk and resilience offer a systematic summary accessible to stakeholders across the professional disciplines and the public alike.

### Challenges of Lifestyle-Based Interventions

“Resilience” is here defined as the comprehensive ability to deal with adversity. Resilience is here not explicitly distinguished from “resistance,” which is often used to specifically identify the ability to ward off pathology. The term “lifestyle factors for risk and resilience” is a heuristic concept to structure the large group of potentially modifiable factors with proven or face value influence on health, that at least in theory can be influenced by the individual by his or her own actions. Actions based on these factors increase or decrease risk and reduce or promote resilience. Low levels of physical activity, for example, are associated with a shorter life expectancy and the increased incidence of cardiovascular disease, cancer, neurodegeneration, and other health issues (Lee et al., 2012); being physically active in turn reduces those risks and prolongs life (Schnohr et al., 2013). This duality might suggest the existence of an equilibrium that could or should be obtained and maintained.

The general usefulness of the term “lifestyle factors” notwithstanding, the heterogeneity of such factors and the conceptual and practical difficulty of capturing what people more broadly mean by “lifestyle” endanger the concept to become either too diffuse or too narrow. While in a scientific study context, lifestyle might have to be reduced to a manageable number of variables that can be measured and that show statistically significant correlations with outcome measures of interest (as well as relevant effect sizes), be it for example longevity or disease-free years, it is not trivial to answer the question of how these variables actually contribute to subjective “lifestyle.” The term “style” refers to this “how” and to rather personal ideas of “leading a good life.” “Lifestyle” is obviously much more than the sum of identifiable “lifestyle factors.”

To understand the impact of lifestyle it is thus not advisable to restrict consideration to only those factors for which the “best” evidence (i.e., large effect sizes in population studies) exists, such as those in the SNAP scheme, which only covers smoking, nutrition, alcohol, and physical activity (Noble et al., 2015). A consensus publication in Lancet Neurology from 2017 highlighted nine factors (Livingston et al., 2017), which essentially match with other consensus lists (Deckers et al., 2015). They are also by and large identical to the set of factors that has been identified for longevity and the prevention of other, mostly age-related diseases, including cancer. The WHO lists eight such general factors plus four more specific to dementia (World Health Organization, 2017). In 2019, the WHO issued guidelines on these factors and supported its recommendations with detailed systematic reviews (World Health Organization, 2019). While this was an important step, the WHO has also been criticized for including recommendations with relatively weak evidence (management of hypertension and diabetes), while omitting other factors with potential benefit and low risks of side effects (treatment of hearing loss and depression) (Lancet, 2019). It was felt that the WHO had missed an opportunity to make even stronger statements for of public health and wake up governments to take the necessary actions. Moreover, in this domain and given the type of existing studies, it is problematic to base recommendations solely on best evidence as generated by clinical studies. The absence of evidence is also here no evidence of absence.

In addition, studies by necessity have to single out identifiable factors for the sake of design, feasibility, and statistical power. There are also first combinatorial and multi-domain studies such as the FINGERS trial (Ngandu et al., 2015) or the SMARTT trial (Yaffe et al., 2018). Even the most comprehensive multidomain study, however, will neither be able to capture the emerging qualities of actual lifestyle nor the one aspect that most people will intuitively value most: that this is their own chosen way of leading their life. The question of how to translate under such conditions from a study setting into everyday life remains extremely challenging.

### The DEEP Framework

**FIGURE 1:**
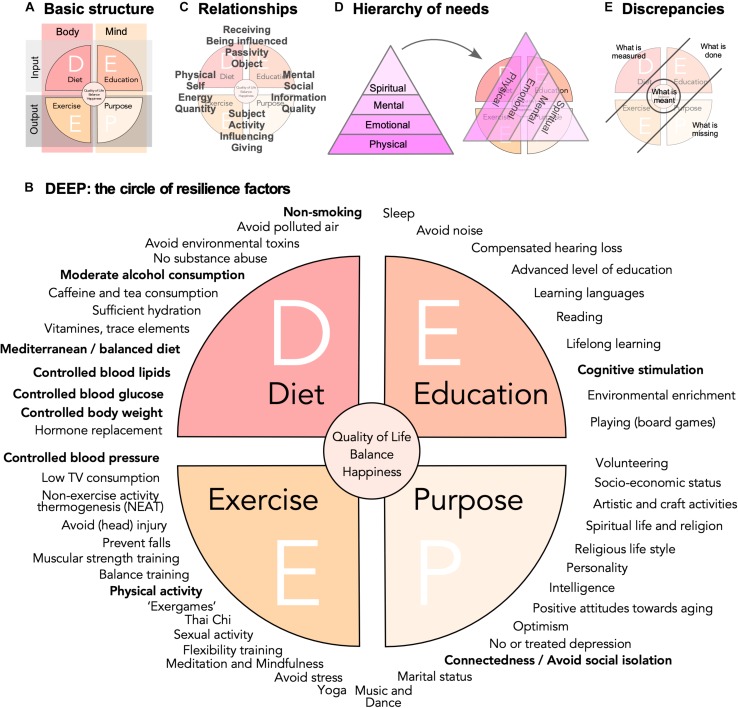
Risk and resilience factors for neurodegenerative disease. **(A)** The DEEP scheme is based on a four-field matrix, spanned out by body and mind as columns, and input and output as rows. **(B)** A non-exclusive list of potential lifestyle risk and resilience factors is represented as a circle with the four quadrants obtained in **(A)**. The WHO factors from the 2019 guidelines are highlighted in bold. They cluster largely in the D quadrant. The WHO in addition states explicitly that vitamin substitution and polyunsaturated fatty acids should not be recommended. Quality of evidence varies greatly, but face validity and popularity might be very high for some factors, for which little data exist. **(C)** The depiction of the DEEP scheme highlights certain relation pairs and tension fields, which are here meant only as illustration of the complex network of relationships that exist and might influence the impact of lifestyle risk and resilience factors and interventions based on them. **(D)** As visualized by a hierarchy of needs that drive motivation, here depicted in a version according to Loehr and Schwartz (2005), people will assign different values to different factors and interventions. **(E)** Further depicts the discrepancy of what is measured (and recommended) and what is done on one side and what is perceived as missing and what is actually meant on the other.

Dependent on stringency and definitions, at least 50 lifestyle risk and resilience factors can be identified (Table A1), that to a variable extent have been discussed in the literature. In this situation of an overabundance of potentially relevant aspects, a simple conceptual framework would facilitate knowledge management, promote sense-making in face of this complexity, and stimulate the development of more sophisticated and causal models across stakeholders, domains, and disciplines. A knowledge management framework can greatly support organization of lifestyle factors in their relations to each other and to larger-scale concepts such as “quality of life,” “well-being,” “happiness,” or, of course, “lifestyle” itself. The DEEP scheme visualizes key relationships between the large number of known (as well as perceived and hypothesized) lifestyle risk and resilience factors (Figures 1A,B).

Basis of the framework is a four-field matrix with *Body* and *Mind* as columns and *Input* and *Output* as rows (Figure 1A). “Diet” (D) would stand for bodily, physical intake and “Education” (E) for sensory, cognitive, and emotional input. “Exercise” (E) represents the domain of physical activity in the broadest sense, while the “Purpose” (P) quadrant is everything that relates between individual’s inner and outer world through his or her own actions. In other words, the four quadrants are related to (Line 1) what we take in (D) physically and (E) cognitively and (Line 2) what we spend (E) physically and (P) cognitively. This captures what essentially all more or less intuitive and research-based advice in this area tells us: Eat well (D), be physically and cognitively active (E+E), and engage (P). Labels for the quadrants and their explanation are emblematic and should not be taken too narrowly: “education,” for example, here stands for mental activity and function in a much broader sense than many scholarly definitions suggest. “Purpose” stands for socially and mentally (or even spiritually) goal-directed activities. The labels are associative anchors, permitting to bring structure into the widest possible scope. It is important not to over-define these categories.

The lifestyle factors, for whose effectiveness we have the best evidence, are some with the highest level of abstraction but with good measurability such as body weight or glucose levels. Education or physical activity cannot be measured directly but usually only be assessed via reductionistic proxies (e.g., VO_2_max for bodily fitness or self-reporting). For some factors in the P quadrant even qualitative proxies are a problem. But the P quadrant contains items that we would consider essentially human and which are determinants of well-being. To base our understanding of lifestyle solely on the relatively few quantifiable factors with “significant” effect sizes is as obviously incomplete as ascribing the genetic risk of complex disease only to the common polymorphisms with large effect sizes. Much like the “missing heritability” in the genetics of complex traits (Manolio et al., 2009), a large number of modifiable factors with small effect sizes and poor detectability will add up to explain a very large part of the total interindividual variance in lifestyle risk and resilience. As much as complex traits are “omnigenic” (Boyle et al., 2017), lifestyle-dependent risk and resilience will be “omni-factorial.”

Appreciating complexity is not *per se* an argument against pursuing lower hanging fruits, i.e., by implementing exercise programs, improving school food, and quit smoking (Norton et al., 2014). These are difficult enough. But while to reduce time spent sitting would be a valuable step forward, regularly standing up at work does not really amount to a different life style. Changing one’s life requires more than working down a check-list of good arguments.

The descriptive inclusiveness is also no argument against applying rigor and the full range of reductionistic instruments of evidence-based medicine to study the individual factors! Those approaches are mandatory for understanding effect sizes, weights of factors, and interaction effects.

## The (P) Purpose Quadrant

Descriptions of lifestyle factors that are centered on diet, exercise, and cognitive activity/education might leave a blind spot exactly where many people would intuitively focus, when asked what matters most in life. Here, social and spiritual aspects rank very high: family and friends as well as mental and religious life, purpose, and autonomy. Certain “lifestyle” risk and resilience factors correspond to this preference: remaining in charge, the role of partnership and friendship, spirituality and religion, taking responsibility for others, etc. Such factors might exert a measurable impact: for example, positive beliefs about aging more or less compensated for the increased risk for AD associated with carrying the ApoEε4 polymorphism (Levy et al., 2018).

The dimension of Purpose-related factors is nevertheless often missing from the high-level aggregation in the discussion of “healthy lifestyle,” presumably because the evidence for these factors does not match the standards (of quantification) in the other domains. But for most people “health” in the sense of the medical professions is no end in itself. They intuitively have a broader, more implicit understanding, seamlessly integrating with “quality of life”: Leading a healthy life is more than the absence of disease. The concept of “successful aging,” otherwise not without its own problems (Bowling and Dieppe, 2005), can add this missing dimension.

## Relationships

In the DEEP scheme, key relationships between domains are emphasized and the communality of lifestyle factors, to which many studies have pointed (Norton et al., 2014), are visible across the entire range of factors, independent of their nature. The scheme is thereby highly interdisciplinary and generous toward different scientific cultures. This is important, because no single discipline covers the full range of contributing factors.

The four quadrants can be paired in various, non-exclusive combinations (Figure 1C). The scheme thereby not only spans out between body and mind and input and output, but at the same time self and social, energy and information, reception and action, quantity and quality, etc. The challenge is to conceptualize, model, and measure such complex relationships and avoid pitfalls (Kempermann, 2017). The secret for livable strategies for successful (cognitive) aging based on lifestyle risk and resilience factors might foremost lie in the individual manifestation of these relationships. Neither represent these ranges dichotomies nor are the tension fields they establish orthogonal to each other. They are rather exemplary forces weaving a matrix of interdependencies.

Some of such interdependencies are known better than others and a few are widely acknowledged: the most obvious might be the relationship between energy intake and physical activity. Many studies calculate communalities, but what these interdependencies ultimately mean is rarely explored, especially for factors that are not linked in a way as obvious as in the case of caloric intake and expenditure. The classical triangle of (Mediterranean) diet, physical exercise, and cognitive activity/education also emphasizes potential links, but takes only three (albeit very important ones) out of an extensive network.

The step from the mere description in the DEEP scheme to a model of the network of factors requires different sets of data than presently available in most cases. Multivariate cohort studies, observational or interventional, can generate such data sets, if they collect information across all four DEEP quadrants.

### The Question of Perspective

The discrepancy between lifestyle in the public-health-centered sense of recommendations and programs for prevention (mostly found in the DEE quadrants) and in what most people would see as integral parts of their personal lifestyle (represented by the P quadrant) might help to explain why the implementation of lifestyle interventions is so difficult. Recommendations based primarily on what can be measured (body weight, blood pressure, caloric intake, VO_2_max, etc.) see lifestyle largely through the filter of basic needs. While these are critical foundations of lifestyle they only partly coincide with our personal hierarchy of needs.

Maslow’s hierarchy of needs, usually displayed in form of a pyramid, remains a very popular means to visualize the essential observation that we differently value the driving forces behind our actions and, by extension, our lifestyle (Maslow, 1954). Various versions exist. Figure 1D depicts a variant from the coaching literature (Loehr and Schwartz, 2005), underscoring that such concepts are widely applied in professions that aim at empowering people to master change. People tend to favor a view that moves from the fulfillment of basic needs toward personal growth, self-actualization, and self-transcendence. While the depiction as pyramid somewhat blurs the fact that motivations arising from many different layers are effective concomitantly and synergistically, its suggestive strengths lie in the identification of an order and a hierarchical value *we assign* to them. A pyramid of needs, tilted by 120°, superimposed on the DEEP scheme, reveals that within the scope of lifestyle factors there is a hidden hierarchy of values.

Recommendations focusing on what can be measured might hence come across as superficial and trivial (Figure 1E). Emphasizing only the P quadrant is no valid solution either, because those strategies lack the concreteness of the physical dimension they have to build upon. But P might provide the drive to implement DEE.

### DEEP Implementations

This implies that a holistic consideration of lifestyle risk and resilience factors along the DEEP scheme might help to develop causal models as basis of individualized strategies for healthy (cognitive) aging and the prevention of (neurodegenerative) disease. Interventions with large demonstrated effect sizes would obviously still be prioritized, but their practical realization and their contextual embedding would be improved by individualized multi-factorial approaches.

Physical activity, for example, remains the preventive “super factor” with massive effect sizes across many domains. Realizing its potential alone would profoundly change health and disease in any population. And most people know that they should (and actually would like to) be more physically active: there is no lack of insight and intentions. The implementation, however, of seemingly simple interventions based on reducing sedentary lifestyle and increasing physical activity is extremely difficult. Exercise-based programs often show remarkably little long-term effects on lifestyle. The same applies to nutritional recommendation, which are popular and often have high face validity. But dietary interventions are notoriously difficult to study, the underlying metabolic mechanisms to which these interventions refer show great inter-individual genetic differences, and eating food is much more than nutrition.

Measures centering only on single or few habits become isolated from the contexts of the individual’s life. New habits that were formed in the lab lack anchoring in everyday life.

Good and bad habits are context-sensitive and circumstances trigger habitual behaviors. Changing habits is difficult, if the world around us reinforces them. The question is, to which extent circumstances can be changed. While there is no general answer to this, there will be room for individual solutions that adapt lifestyle to conditions at hand and explore the available room for development. The DEEP scheme visualizes the scope of such co-factors.

### What Lies at the Center?

The large cohort studies and meta-analyses indicate that the identifiable factors with large effect sizes are not independent of each other. On one side this suggests that an abstract “super factor” might be identifiable, comparable to the G factor in intelligence.

On the other hand, however, the communalities also indicate that there is more than one road to Rome. They might hide the range of options for the individual to achieve his or her goals.

The DEEP scheme might suggest that the greatest reduction in risk and the greatest level of resilience is found in individuals with a somewhat balanced nature within and between the four quadrants (Figure 1E). How would such balance relate to “quality of life”? While assessment of quality of life constitutes a major achievement in selecting appropriate, relevant “endpoints” of clinical studies, they are only surrogates for even more profound constructs such as happiness. Is a resilient individual also a happier individual? If so, what would be the direction of causality here? Positive psychology suggests that happiness can to some degree be induced. If “style” is an emerging property from the complexity of life, then so is happiness. An important question thus is, whether the assumed causality structure and interventional strategy can be put on its head. Is it possible to improve happiness and quality of life and see changes in measurable lifestyle factors as consequence or byproduct? Or, is the true center an attitude such as to “*develop character in the face of the inevitable suffering*,” as Peterson (2018) has put it, and thereby consider style of life in the light of giving meaning to life?

Working from the center and the intended result of “leading a good life,” including the full spectrum of our needs and values, is one way of altering contexts to our benefit. Ironically, despite being much more complex, multi-factorial changes might be more successful and ultimately easier: small steps at a time, but along different directions (as indicated in the many options of the DEEP scheme), might ultimately be more efficient and effective than focusing on one high-gain domain alone. They would lead to a diversification of the individual portfolio of lifestyle risk and resilience.

## Data Availability

No datasets were generated or analyzed for this study.

## Author Contributions

GK conceived the concept presented in this article, collected the relevant information, and wrote the manuscript.

## Conflict of Interest Statement

The author declares that the research was conducted in the absence of any commercial or financial relationships that could be construed as a potential conflict of interest.

## APPENDIX

**TABLE A1:** The table lists potential lifestyle resilience factors for neurodegenerative disease, independent of whether the cited publications are referring to the risk or resilience side of the factor.

		**Quote from the report**	**Type of study**	**References**	**Remarks**
**D Diet**					
**Non-smoking**	+	Smoking is associated with an increased risk of dementia.	Meta-analysis	Zhong et al., 2015	
	+	Elderly smokers have increased risks of dementia and cognitive decline.	Meta-analysis of prospective studies	Anstey et al., 2007	
Avoid environmental risk factors	(+)	There is at least moderate evidence implicating the following risk factors: air pollution, aluminum, silicon, selenium, pesticides, vitamin D deficiency, and electric and magnetic fields.	Systematic review	Killin et al., 2016	
Avoid air pollution from traffic	+	Living close to heavy traffic was associated with a higher incidence of dementia, but not with Parkinson’s disease or multiple sclerosis.	Population-based cohort, *n* = 6.6 mio	Chen et al., 2017	
No substance abuse	(+)	Hyperphosphorylated tau and amyloid precursor protein deposition is increased in the brains of young drug abusers.	Post-mortem case–control study	Ramage et al., 2005	
	(+)	Former use of benzodiazepines could be a risk factor for dementia.	Nested case–control study	Lagnaoui et al., 2002	
	+	Benzodiazepine use is associated with an increased risk of Alzheimer’s disease (AD).	Case–control study	Billioti de Gage et al., 2014	
	+	Out of the 10 studies retrieved, 9 reported an increased risk of dementia in benzodiazepine users.	Systematic review	Billioti de Gage et al., 2015	
**Moderate alcohol consumption**	+	High proportion of alcohol-related dementia in early-onset dementia.	Systematic review	Cheng et al., 2017	
	+	Middle-aged adults with a history of alcohol use disorders have increased odds of developing severe memory impairment later in life.	Prospective cohort study (19 years, 6500 participants)	Kuźma et al., 2014	
	+	Alcohol use disorders were a major risk factor for onset of all types of dementia, and especially early-onset dementia.	Retrospective cohort study (20 years, 31 Mio. patients)	Schwarzinger et al., 2018	
	+	The results show a greater risk of dementia in those who abstain from alcohol or consume >14 units/week, with risk increasing in a linear fashion at higher levels of consumption.	Prospective cohort study (23 years, *n* = 9000)	Sabia et al., 2018	One of the key studies supporting the idea that low alcohol consumption might be beneficial.
**Caffeine and tea consumption**	(+)	Several cross-sectional and longitudinal population-based studies suggested a protective effect of coffee, tea, and caffeine use against late-life cognitive impairment/decline.	Systematic review	Panza et al., 2015	
	(+)	Daily tea drinking is associated with decreased risk of cognitive impairment, MCI, and cognitive decline in the elderly. However, the association between tea intake and AD remains elusive	Meta-analysis, 52,503 participants	Ma et al., 2016	
Sufficient hydration^*^	+/–	Inconsistent evidence of the impact of dehydration on cognitive performance. Current findings in the field suggest that particular cognitive abilities and mood states are positively influenced by water consumption.	Review	Masento et al., 2014	Drinking sufficient amounts of water is a popular advice, but backed by limited evidence. Because dehydration promotes delirium and thus can aggravate dementia, especially in care settings, this factor nevertheless has good face validity.
Vitamins, trace elements, antioxidants	?	Value of vitamin D substitution in cognitive impairment remains doubtful.	Systematic literature research and meta-analysis	Etgen et al., 2011	
	+	Within the range of a habitual dietary intake, higher intake of lignans is associated with less cognitive decline at middle age.	Retrospective cohort study	Nooyens et al., 2015	Example of the large class of polyphenols.
	?	Findings do not consistently show habitual intakes of dietary antioxidants and are associated with better cognitive performance or a reduced risk for dementia.	Systematic review	Crichton et al., 2013	
	+	Vitamin E intake, from foods or supplements, is associated with less cognitive decline with age. There was little evidence of association with vitamin C or carotene intake.	Longitudinal population-based study	Morris et al., 2002	The WHO found the available evidence sufficient to recommend against the use of vitamin substitution in its 2019 guidelines.
**Medit./balanced diet**	+	In an older population, a Mediterranean diet supplemented with olive oil or nuts is associated with improved cognitive function.	Randomized clinical trial	Valls-Pedret et al., 2015	
	+	Pooled results suggest that a higher adherence to the MeDi is associated with a reduced risk of developing MCI and AD, and a reduced risk of progressing from MCI to AD.	Systematic review	Singh et al., 2014	
**Controlled blood lipids**	+	Significant gaps in the literature regarding total cholesterol and late-life dementia remain. Evidence suggests that high midlife total cholesterol increases risk of late-life AD, and may correlate with the onset of AD pathology.	Meta-analysis, *n* = 23,338	Anstey et al., 2017	
**Controlled glucose metabolism**	+	Higher glucose levels may be a risk factor for dementia, even among persons without diabetes.	Prospective study	Crane et al., 2013	
**Controlled body weight**	+	Underweight, overweight, and obesity in midlife increase dementia risk.	Meta-analysis of 15 prospective studies, *n* = 15,435	Anstey et al., 2011	
	+	Studies investigating the association between midlife BMI and risk for dementia demonstrated generally an increased risk among overweight and obese adults. When measured in late-life, elevated BMI has been associated with lower risk. In addition, being underweight and/or having a decrease in BMI in late-life are associated with higher dementia risk compared to BMI in the normal range or stable BMI.	Review	Emmerzaal et al., 2015	
	+	Being underweight in middle age and old age carries an increased risk of dementia over two decades.	Retrospective cohort study, *n* = 2 million	Qizilbash et al., 2015	Controversial study that contradicts some conclusions from other studies in that no effect of overweight was reported.
Hormone replacement	–	Estrogen plus progestin therapy increased the risk for probable dementia in postmenopausal women aged 65 years or older. In addition, estrogen plus progestin therapy did not prevent mild cognitive impairment in these women. These findings, coupled with previously reported WHI data, support the conclusion that the risks of estrogen plus progestin outweigh the benefits.		Shumaker et al., 2003	There is still considerable controversy regarding this conclusion, but there is no clear evidence in favor of the idea that hormone therapy lowers the risk for dementia.
**E Exercise**					
**Controlled blood pressure**	+/−	There is no convincing evidence from the trials identified that blood pressure lowering in late-life prevents the development of dementia or cognitive impairment in hypertensive patients with no apparent prior cerebrovascular disease.	Meta-analysis (Cochrane), *n* = 15,936	McGuinness et al., 2009	No clear case can yet be made for interventions at older age.
	+	Elevated blood pressure during midlife, persistence of elevated blood pressure into late life, and, among non-hypertensives, a steep decline in blood pressure during mid- to late-life were associated with an increased dementia risk in a community-based cohort. Our data highlight the potential sustained cognitive benefits of lower blood pressures in mid-life and also suggest that declining blood pressure in older adults with prehypertension or normotension, but not in those with hypertension, may be a risk marker for dementia.	Retrospective study, *n* = 1440	McGrath et al., 2017	This study highlights the complex relationship between control of blood pressure and the risk of dementia.
	+	In this cohort of older adults, increased numbers of optimal cardiovascular health metrics and a higher cardiovascular health score were associated with a lower risk of dementia and lower rates of cognitive decline. These findings may support the promotion of cardiovascular health to prevent risk factors associated with cognitive decline and dementia.	Population-based cohort study; *n* = 6626	Samieri et al., 2018	
Reduce TV consumption	+	High television viewing and low physical activity in early adulthood were associated with worse midlife executive function and processing speed.	Prospective 25-year study	Hoang et al., 2016	TV consumption represents the largest part of average sedentary behaviors.
Non-exercise activity thermogenesis (NEAT)^*^	(+)	NEAT is a highly variable component of daily total energy expenditure and a low level of NEAT is associated with obesity. NEAT enhances lifestyle, and variations in individual and environmental factors can significantly affect daily energy expenditure.	Review	Chung et al., 2018	No data directly related to neurodegeneration and brain function exist.
Avoid head injury	+	Although further research is needed, these 10 findings suggest that dose-dependent effects of violent head displacement in vulnerable brains predispose to dementia.	Review	Mendez, 2017	
	(+)	Meta-analysis revealed a significant association of prior TBI with subsequent dementia.	Meta-analysis, *n* = 3,263,207	Huang et al., 2018	The authors come to the opposite conclusion because the effect here disappeared for individual neurodegenerative diseases (Simpson’s paradox).
	+	History of TBI, including mild TBI, is associated with the development of neurological and psychiatric illness. This finding indicates that either TBI is a risk factor for heterogeneous pathological processes or that TBI may contribute to a common pathological mechanism.	Meta-analysis	Perry et al., 2016	Increased pooled odds ratios for “dementia,” Parkinson’s disease, AD, and other disorders.
Prevent falls^*^	(+)	n/a	n/a	n/a	Preventing the disabling consequences of falls supports the positive effects of physical activity. Currently no studies exist on falls as modifiable risk factor for neurodegeneration.
Balance training	(+)	Balance training intervention […] was effective in improving balance and mobility, […]. Cognition did not decline during the course of the intervention but did decline following the intervention, suggesting a possible protective effect.	Prospective study	Ries et al., 2015	
**Physical activity**	+	Meta-analyses of prospective studies documented a significantly reduced risk of dementia associated with midlife exercise; similarly, midlife exercise significantly reduced later risks of mild cognitive impairment in several studies.	Review of meta-analyses and other reports	Ahlskog et al., 2011	
	+	We found a higher level of physical activity to be associated with a lower risk of dementia.	Prospective, population-based study (Rotterdam Study), *n* = 4406	de Bruijn et al., 2013	One of the key long-term population studies, supporting the claim.
	+	This meta-analysis suggests that physical activity interventions positively influence cognitive function in patients with dementia.	Meta-analysis, *n* = 802	Groot et al., 2016	
Muscular strength training	(+)	All studies concluded that cognitive function and grip strength declined, on average, with increasing age, although with little to no evidence for longitudinal associations among rates of change.	Systematic review	Zammit et al., 2018	
	+	High-intensity progressive resistance training results in significant improvements in cognitive function, muscle strength, and aerobic capacity in older adults with MCI. Strength gains, but not aerobic capacity changes, mediate the cognitive benefits of progressive resistance training.	Randomized, double-blind, double-sham controlled trial; *n* = 100	Mavros et al., 2017	
	+	Resistance training significantly improved global cognitive function, with maintenance of executive and global benefits over 18 months.	Randomized, double-blind, double-sham controlled trial; *n* = 100	Fiatarone Singh et al., 2014	
Exergames (physically active video games)		Exergames significantly improved global cognition.	Meta-analysis of 17 randomized controlled trials, *n* = 926	Stanmore et al., 2017	Control groups are physically active.
Walking groups^*^	(+)	Walking groups are effective and safe with good adherence and wide-ranging health benefits.	Systematic review and meta-analysis, 1843 patients, including PD patients	Hanson and Jones, 2015	Outcome measure: general health, depression score, VO2max. It is unclear, at present, how much of the observed effect can be attributed to physical activity *per se* rather than the social aspect of this activity.
Flexibility training^*^		n/a	n/a	n/a	Flexibility is one of the three pillars of physical activity (besides endurance and strength), but its contribution to prevention of dementia and neurodegeneration has not yet been studied.
Tai Chi	(+)	Compared with usual physical activities, Tai Chi shows potential protective effects on healthy adults’ cognitive ability.	Systematic review of prospective controlled trials, *n* = 632	Zheng et al., 2015	
Sexual activity^*^	(+)	There were significant associations between sexual activity and number sequencing and recall in men.	Cross-sectional study, *n* = 6833, two tests of cognitive functioning	Wright and Jenks, 2016	Protective effects of sexual activity are a popular assertion in the media with a certain face validity and some evidence from studies, but currently still backed by insufficient data.
	(+)	After controlling for demographic and health-related lifestyle factors, more frequent sexual activity and greater emotional closeness during partnered sexual activity were associated with better memory performance.	Prospective study, *n* = 6016	Allen, 2018	
Meditation and mindfulness	(+)	All studies reported significant increases in gray matter volume in the meditators/intervention group, albeit in assorted regions of the brain. Limited research exists on the mechanisms through which meditation affects disease-related neurodegeneration, but preliminary evidence suggests that it may offset gray matter atrophy.	Review	Last et al., 2017	
Avoid stressors	(+)	Higher event-based stress ratings collected over the follow-up period were associated with faster cognitive decline in subjects with mild cognitive impairment but not in cognitively normal subjects.	Prospective study, *n* = 52	Peavy et al., 2009	
Yoga	(+)	Based on the available literature, it could be concluded that yoga might be considered as an effective adjuvant for the patients with various neurological disorders.	Review	Mooventhan and Nivethitha, 2017	
Music and dance	+	Playing a musical instrument was significantly associated with less likelihood of dementia and cognitive impairment.	Twin study, *n* = 157 twins	Balbag et al., 2014	
	+	Recent and past musical activity, but not general lifestyle activities, predicted variability across both verbal and visuospatial domains in aging. […] Early age of musical acquisition, sustained and maintained during advanced age, may enhance cognitive functions and buffer age and education influences.	Cross-sectional case control study, *n* = 70	Hanna-Pladdy and Gajewski, 2012	
	+	Among leisure activities, reading, playing board games, playing musical instruments, and dancing were associated with a reduced risk of dementia.	Prospective cohort, *n* = 469	Verghese et al., 2003	
E Education					
Sleep	+	Sleep disturbances may predict the risk of incident dementia.	Systematic review and meta-analysis, *n* = 246,786	Shi et al., 2018	
Avoid noise	(+)	Increases in dementia risk were also observed with […] night-time noise levels.	Retrospective cohort study, *n* = 130,978	Carey et al., 2018	The association did not remain significant in multipollutant models.
Compensated midlife hearing loss	?	When completed in 2022, Aging and cognitive health evaluation in elders study should provide definitive evidence of the effect of hearing treatment versus education control on cognitive decline in community-dwelling older adults with mild-to-moderate hearing impairment.	Multicenter randomized controlled trial, *n* = 850	Deal et al., 2018	Ongoing trial.
	+	Hearing loss is independently associated with accelerated cognitive decline and incident cognitive impairment in community-dwelling older adults.	Prospective observational study, *n* = 1984	Lin et al., 2013	The WHO found the available evidence insufficient to recommend treating hearing loss in its 2019 guidelines.
	+	Comparing participants with moderate/severe hearing impairment to participants with no hearing impairment, 20-year rates of decline in memory and global function differed by −0.47 standard deviations (*P* = 0.02) and −0.29 standard deviations (*P* = 0.02), respectively. Estimated declines were greatest in participants who did not wear a hearing aid.	Cross-sectional and longitudinal analysis in a prospective study, *n* = 253	Deal et al., 2015	
Level of education	(+)	Lower education was associated with a greater risk for dementia in many but not all studies. The level of education associated with risk for dementia varied by study population and more years of education did not uniformly attenuate the risk for dementia. It appeared that a more consistent relationship with dementia occurred when years of education reflected cognitive capacity, suggesting that the effect of education on risk for dementia may be best evaluated within the context of a lifespan developmental model.	Systematic review	Sharp and Gatz, 2011	
Languages	+	Lifelong bilingualism confers protection against the onset of AD.	Retrospective study on AD patients, *n* = 211	Craik et al., 2010	Childhood bilingualism, which has been assessed here, is not identical to the popular assertion that learning a new language later in life is protective!
	+	Our results suggest a positive effect of bilingualism on later−life cognition, including in those who acquired their second language in adulthood.	Population-based cohort study, *n* = 853	Bak et al., 2014	Sixty-five participants learned the second language after the age of 18 years.
	(+)	We did not find that bilingualism protects from cognitive decline or dementia from prospective studies.	Systematic review and meta-analysis	Mukadam et al., 2017	The conclusion is controversial, because the retrospective studies in the analysis did show the association.
Reading	+	Among leisure activities, reading, playing board games, playing musical instruments, and dancing were associated with a reduced risk of dementia.	Prospective cohort, *n* = 469	Verghese et al., 2003	
Lifelong learning	+	More frequent cognitive activity across the life span has an association with slower late-life cognitive decline.	Longitudinal cohort study, *n* = 294	Wilson et al., 2013	
Cognitive stimulation	+	Frequent participation in cognitively stimulating activities is associated with reduced risk of AD	Longitudinal cohort study (“Nun study”), *n* = 801	Wilson et al., 2002	Screened activities included: viewing television; listening to radio; reading newspapers; reading magazines; reading books; playing games such as cards, checkers, crosswords, or other puzzles; and going to museums.
Environmental enrichment	+	The longitudinal evidence consistently shows that engaging in intellectually stimulating activities is associated with better cognitive functioning at later points in time.	Review	Hertzog et al., 2008	Extensive review of the overarching concept, comprising many aspects covered elsewhere here.
Playing board games	+	Among leisure activities, reading, playing board games, playing musical instruments, and dancing were associated with a reduced risk of dementia.	Prospective cohort, *n* = 469	Verghese et al., 2003	
**P Purpose**					
Volunteering	(+)	Cohort studies showed volunteering had favorable effects on depression, life satisfaction, wellbeing but not on physical health. These findings were not confirmed by experimental studies. Meta-analysis of five cohort studies found volunteers to be at lower risk of mortality.	Systematic review	Jenkinson et al., 2013	
	(+)	Our results largely support the assumptions that voluntary work in later life is associated with lower self-reported cognitive complaints and a lower risk for dementia, relative to those who do not engage, or only engage episodically in voluntary work.	Population-based cohort study (registry study), *n* = 1001)	Griep et al., 2017	
	+	Volunteering at the initial assessment and volunteering regularly over time independently decreased the risk of cognitive impairment over 14 years, and these findings were maintained independent of known risk factors for cognitive impairment.	Panel survey, *n* = 13,262	Infurna et al., 2016	
Socio-economic status	+	Significant interactions were found between a healthful lifestyle (defined as having more than or equal to three healthful lifestyle factors) and income on changes of global cognition and verbal fluency […]. The protective effect of a healthful lifestyle was observed only among participants with lower income in global cognition and logical memory […].	Prospective cohort study, *n* = 603	Weng et al., 2018	
	+/−	Occupational social class was not statistically significantly associated with dementia death in men or women.	Meta-analysis, of 11 prospective cohort studies, *n* = 86,508	Russ et al., 2013	
Artistic and craft activities	+	The risk [of MCI] was reduced with engagement in artistic, craft, and social activities in both midlife and late life.	Population-based prospective cohort study, *n* = 256	Roberts et al., 2015	
Spiritual life and religion	(+)	Study results show an inverse association between religious attendance in 1982 and cognitive dysfunction in 1985.	Population-based cohort study, *n* = 2812	Van Ness and Kasl, 2003	
	(+)	Religious attendance may offer mental stimulation that helps to maintain cognitive functioning in later life, particularly among older depressed women.	Population-based cohort, study, *n* = 2938	Corsentino et al., 2009	
	+	Spirituality and religion appear to slow cognitive decline, and help people use coping strategies to deal their disease and have a better quality of life.	Systematic review	Agli et al., 2015	
Religious life-style	(−)	The prevalence of dementia was increased among men with exclusively religious education and among those with the most strict observance. In both cases, these associations were not altered appreciably after controlling for sociodemographic confounders.	Longitudinal cohort study (Israeli Ischemic Heart Disease study), *n* = 1628	Beeri et al., 2008	
Personality	(+)	Using brief assessments of personality and cognition, we found robust evidence that personality is associated with risk of cognitive impairment and dementia in a large national sample.	Population-based, longitudinal study, *n* = 10,000	Terracciano et al., 2017	Personality traits are to some extent amenable to volitional change (Roberts et al., 2017).
	+/−	Neuroticism increased risk for dementia, and conscientiousness reduced risk. The protective effect of openness was tentative. Extraversion and agreeableness were not associated with dementia.	Systematic review and meta-analysis, *n* = 3285	Low et al., 2013	
Intelligence	+	Compared to the highest intelligence group (≥115), dementia risk was raised in the lowest-scoring category (<85) and these associations were stronger for women […] than men […]. There was evidence of a dose–response association between childhood IQ and dementia in women […] but not in men […].	Population-based cohort study (1932 Scottish Mental Survey), *n* = 32,000	Russ et al., 2017	Relevance of intelligence as modifiable risk and resilience factor relies on the question, whether intelligence can be improved, which despite many claims to the contrary remains questionable (Haier, 2014).
Positive attitudes toward aging	+	In the total sample those with positive age beliefs at baseline were significantly less likely to develop dementia, after adjusting for relevant covariates. Among those with APOE ε4, those with positive age beliefs were 49.8% less likely to develop dementia than those with negative age beliefs.	Prospective cohort study	Levy et al., 2018	
Optimism	+	Optimism was prospectively associated with a reduced likelihood of becoming cognitively impaired.	Prospective cohort study, *n* = 9568	Gawronski et al., 2016	
No or treated depression	+	The literature suggests an association between depression and dementia, and growing evidence implies that timing of depression may be important to defining the nature of the association.	Review	Byers and Yaffe, 2011	The WHO found the available evidence insufficient to recommend treating depression in its 2019 guidelines.
	−	Depressive symptoms in the early phase of the study corresponding to midlife, even when chronic/recurring, do not increase the risk for dementia. Along with our analysis of depressive trajectories over 28 years, these results suggest that depressive symptoms are a prodromal feature of dementia or that the two share common causes. The findings do not support the hypothesis that depressive symptoms increase the risk for dementia.	Population-based cohort study (Whitehall II), *n* = 10,308	Singh-Manoux et al., 2017	
Connected-ness, avoid social isolation	+	Living alone [relative risk ratio (RRR) = 5.814; *p* = 0.000] and self-reported loneliness (RRR = 1.928, *p* = 0.049) were associated with a greater risk of cognitive difficulty. Living arrangements, perceived social support, and loneliness were found to moderate the relationship between the APOE e4 allele and cognitive function.	Cross-sectional study, *n* = 779	Poey et al., 2017	The WHO found the available evidence insufficient to recommend social activity in its 2019 guidelines.
	+	Feeling lonely rather than being alone is associated with an increased risk of clinical dementia in later life and can be considered a major risk factor.	Prospective cohort study, *n* = 2173	Holwerda et al., 2014	
Marital status	+	We confirmed an association between marital status and AD, with an excess risk observed among never-married individuals.	Population-based cohort study, *n* = 3675	Helmer et al., 1999	
	+	Those living alone as non-marrieds may be at risk for early-onset and late-onset dementia.	Prospective population-based study, *n* = 31,572	Sundström et al., 2016	

*In a few cases, references have been included that do not specifically deal with neurodegeneration, if the factor has been suggested in the context but no published study exists. The table provides an annotated reference list to the DEEP scheme and does not represent a systematic review of the subject. Bold text: modifiable risk factors according to WHO guideline (2019). The WHO publication also contains a systematic review of the evidence for these factors. ^*^, factors with face validity that are found in popular media or are suggested by context; +, the literature supports a positive association; −, the literature supports a positive association; (+), the literature suggests a positive association but is not fully conclusive; (−), the literature suggests a negative association but is not fully conclusive; +/–, the literature indicates a variable association or neutrality; ?, evidence cannot be conclusively judged at this time.*
